# Nanoparticle-Based Delivery of Resveratrol Suppresses Ehrlich Ascites Carcinoma and Protects Testicular Function via Antioxidant, Anti-Angiogenic, Anti-Inflammatory, and Pro-Apoptotic Mechanisms

**DOI:** 10.3390/biom15111605

**Published:** 2025-11-15

**Authors:** M. Alfawaz, Ekramy M. Elmorsy, Ahmad Najem Alshammari, Marwa Nagy Emam, Islam Ibrahim Hegab, Aly A. M. Shaalan, Manal S. Fawzy, Lina Abdelhady Mohammed

**Affiliations:** 1 Department of Medical Laboratory Technology, College of Applied Medical Sciences, Northern Border University, Arar 91431, Saudi Arabia; mohammed.alfawaz@nbu.edu.sa (M.A.); ahmad.alshammari2@nbu.edu.sa (A.N.A.); 2 Center for Health Research, Northern Border University, Arar 73213, Saudi Arabia; ekramy.elmorsy@nbu.edu.sa; 3 Department of Forensic Medicine and Clinical Toxicology, Faculty of Medicine, Mansoura University, Mansoura 35516, Egypt; 4 Physiology Department, Faculty of Medicine, Tanta University, Tanta 31511, Egypt; marwa2024@ibnsina.edu.sa (M.N.E.); dr.islamhegab@ibnsina.edu.sa (I.I.H.); 5 Department of Biophysiology, Ibn Sina National College for Medical Studies, Jeddah 22413, Saudi Arabia; 6 Department of Anatomy, Faculty of Medicine, Jazan University, Jazan 45142, Saudi Arabia; ashaalan@jazanu.edu.sa; 7 Department of Histology and Cell Biology, Faculty of Medicine, Suez Canal University, Ismailia 41522, Egypt; 8 Department of Medical Biochemistry and Molecular Biology, Faculty of Medicine, Benha University, Benha 13518, Egypt; lina.mohamed@fmed.bu.edu.eg

**Keywords:** resveratrol-loaded nanoparticles, Ehrlich ascites carcinoma, testicular function, anti-angiogenic, antioxidant, anti-inflammatory, apoptosis

## Abstract

This study, for the first time, evaluated the therapeutic potential of resveratrol-loaded phytosome nanoparticles (RES-PNPs) against Ehrlich ascites carcinoma (EAC) and associated testicular dysfunction, compared with free resveratrol (RES). Ninety male Swiss albino mice were divided into six groups, (1) control; (2) RES (10 mg/kg/day, orally); (3) RES-PNPs (10 mg/kg/day, orally); (4) EAC, induced by intraperitoneal injection of 2.5 × 10^6^ cells; (5) EAC + RES; and (6) EAC + RES-PNPs, treated for 20 days post-tumor inoculation. Tumor growth parameters, reproductive function, antioxidant enzyme activities, inflammatory mediators, apoptotic markers, and histopathological features were assessed. Additionally, in silico docking was performed to identify molecular targets mediating RES effects. RES-PNPs markedly reduced tumor volume, ascitic cell viability, and body weight gain while significantly prolonging survival compared with free RES. Molecular assays revealed enhanced pro-apoptotic signaling (increased Bax and Caspase-3, decreased Bcl-2), suppression of vascular endothelial growth factor (VEGF), and inhibition of COX-2 with reduced TNF-α, IFN-γ, and IL-1β levels. RES-PNPs also restored semen quality, normalized reproductive hormones, elevated antioxidant enzyme activities, and reduced lipid and protein oxidation, corroborated by notable testicular histological protection. In conclusion, Resveratrol-loaded phytosome nanoparticles provide superior anti-tumor, antioxidant, anti-inflammatory, and pro-apoptotic benefits compared with free RES. These findings highlight RES-PNPs as a potent and stable nanoformulation for effective EAC suppression and preservation of male reproductive integrity.

## 1. Introduction

Cancer is a multifactorial and progressive disorder arising from disruptions in cellular signaling pathways that control proliferation, differentiation, and homeostasis, typically triggered by genetic mutations and oncogenic drivers [1]. Tumor development proceeds through sequential stages characterized by hallmark features, including resistance to antiproliferative signals, uncontrolled cell division, replicative immortality, angiogenic stimulation, evasion of apoptosis, and the capacity for invasion and metastasis [2]. The metastatic cascade depends on angiogenesis for the continuous supply of oxygen and nutrients, a process regulated by a delicate equilibrium between pro-angiogenic and anti-angiogenic factors [3]. Among these, vascular endothelial growth factor (VEGF) is a master regulator that stimulates neovascularization and increases vascular permeability, thereby facilitating tumor growth and the formation of malignant ascites [4].

Malignant ascites, characterized by abnormal accumulation of fluid in the peritoneal cavity, is a frequent manifestation of advanced intra-abdominal malignancies [5]. It arises primarily from tumor-induced lymphatic obstruction, excessive vascular permeability, and enhanced angiogenesis [6]. In the Ehrlich ascites carcinoma (EAC) model, tumor cells proliferate freely within the peritoneal cavity, producing oxidative stress, inflammatory cytokines, and pro-angiogenic mediators that promote aggressive tumor expansion and dissemination to distant organs, including the liver, kidneys, and spleen [7]. Beyond its oncogenic potential, EAC has been documented to exert systemic deleterious effects that extend to the male reproductive system. Several studies have demonstrated that EAC-bearing male mice exhibit significant testicular degeneration, impaired spermatogenesis, and decreased serum testosterone levels due to elevated reactive oxygen species (ROS) production, lipid peroxidation, and activation of pro-inflammatory mediators [8,9]. These pathological alterations suggest that EAC induces male reproductive dysfunction through oxidative and endocrine disruption. Therefore, assessing testicular parameters alongside tumor progression provides an integrated understanding of cancer-induced systemic toxicity. Based on this rationale, evaluating both the anti-cancer efficacy and the potential testicular protective effects of novel therapeutics, such as resveratrol-based nanocarriers, is of great scientific importance. Moreover, EAC-induced systemic oxidative stress and inflammation exert secondary toxic effects on testicular tissue, impairing spermatogenesis and hormonal balance [10].

Despite advances in modern anti-cancer modalities such as chemotherapy, radiotherapy, immunotherapy, and molecularly targeted drugs, their application remains constrained by severe systemic toxicity, nonspecific cytotoxicity, high cost, and the eventual emergence of multidrug resistance [11]. These limitations have catalyzed a growing interest in plant-derived bioactive molecules that offer multitargeted pharmacological actions with acceptable safety profiles [12,13].

Resveratrol (RES; trans-3,4,5-trihydroxystilbene; PubChem CID:445154) (https://pubchem.ncbi.nlm.nih.gov/compound/Resveratrol) (last accessed on 26 October 2025) is a natural polyphenolic compound found abundantly in grapes, peanuts, and cranberries. It has gained substantial attention for its potent antioxidant, anti-inflammatory, and chemopreventive effects [14]. Extensive in vitro and in vivo studies have demonstrated its capacity to inhibit tumor initiation and progression through multiple mechanisms, including COX-2 inhibition [15], modulation of 5-lipoxygenase signaling [16], and induction of apoptosis via Bax upregulation and caspase activation [17]. Beyond its anti-cancer effects, RES exerts beneficial actions on male reproductive health by improving sperm motility, count, and DNA integrity, primarily through activation of the AMPK pathway and attenuation of oxidative stress [18,19]. Moreover, clinical findings indicate that RES supplementation improves sperm parameters in patients with idiopathic infertility without adversely affecting seminal characteristics [19,20].

Nevertheless, the therapeutic exploitation of RES is greatly restricted by its low aqueous solubility, rapid metabolism, limited membrane permeability, and poor systemic bioavailability [21]. To overcome these pharmacokinetic barriers, nanotechnology-based drug delivery systems have been developed. Among them, phytosome nanoparticles, composed of phospholipid bilayer vesicles, offer a superior platform capable of encapsulating both hydrophilic and lipophilic compounds, thereby improving solubility, membrane permeability, and bioavailability while providing sustained release and enhanced stability [22].

Encapsulation of RES into phytosome nanoparticles (RES-PNPs) is therefore expected to amplify its therapeutic profile while preserving its inherent antioxidant and anti-inflammatory activities. Based on this rationale, the present study was designed to investigate, for the first time, the anti-tumor efficacy of RES-PNPs against Ehrlich ascites carcinoma and their potential to mitigate associated testicular injury. We hypothesized that RES-PNPs exert synergistic anti-cancer effects through antioxidant, anti-angiogenic, anti-inflammatory, and pro-apoptotic mechanisms, while simultaneously protecting testicular tissue from oxidative and inflammatory damage induced by EAC progression.

## 2. Materials and Methods

### 2.1. Molecular Docking

Molecular docking analyses were performed to predict the interactions between resveratrol and key antioxidant and pro-inflammatory target proteins using the SwissDock web server (http://www.swissdock.ch/) (last accessed on 28 October 2025). The molecular targets included catalase (CAT), superoxide dismutase (SOD), glutathione peroxidase (GSH-Px), tumor necrosis factor-alpha (TNF-α), interleukin-1β (IL-1β), interferon-gamma (IFN-γ), and cyclooxygenase-2 (COX-2). The three-dimensional structure of resveratrol was retrieved from PubChem, and its geometry was energy-minimized using Open Babel software 3.1.1 to optimize the ligand for docking. Crystal structures of the selected proteins were obtained from the Protein Data Bank (PDB), and pre-processing steps included removing non-essential water molecules and ligands from the protein structures using established molecular visualization tools. Docking simulations were executed in the Accurate mode of SwissDock, which utilizes the EADock DSS engine and AutoDock Vina scoring function to generate and rank binding conformations based on predicted binding affinity (ΔG, kcal/mol). For each protein–ligand complex, the most favorable binding poses and minimum free energy scores were identified. The SwissDock graphical interface facilitated comprehensive visualization of ligand–protein interactions, enabling assessment of hydrogen bonds, hydrophobic contacts, ionic interactions, and key amino acid residues involved in complex stabilization. All docking analyses were conducted with default parameters unless otherwise stated, and results were interpreted in the context of potential biological relevance.

### 2.2. Preparation of Resveratrol-Loaded Phytosome Nanoparticles (RES-PNPs)

Resveratrol-loaded phytosome nanoparticles (RES-PNPs) were prepared using a modified thin-layer hydration technique, previously reported for the synthesis of phytosomal nanocarriers [22]. The formulation comprised RES, phosphatidylcholine, and cholesterol at optimized molar ratios (RES: Phosphatidylcholine 1:2, cholesterol 5–10% *w*/*w* of total lipids) to ensure structural stability and sustained bioactivity. Phosphatidylcholine served as the primary carrier lipid owing to its amphiphilic nature, which improves both hydrophilic and lipophilic drug encapsulation, facilitates intestinal absorption, and enhances phytosome stability. Cholesterol (5–10%, *w*/*w* of total lipids) was incorporated to strengthen the bilayer structure, minimize drug leakage, and improve dispersion stability. RES was dosed at 25 mg/kg, using a 1:2 molar ratio with phosphatidylcholine to optimize entrapment efficiency and particle homogeneity.

For preparation, phosphatidylcholine (100 mg) and RES (50 mg) were dissolved in 10 mL of methanol, while cholesterol (10 mg) was dissolved separately in 5 mL of dichloromethane. The two organic phases were mixed in a round-bottom flask and evaporated under reduced pressure using a rotary evaporator (Heidolph, Schwabach, Germany) followed by probe sonication using a Sonics Vibra-Cell device (Sonics & Materials, Inc., Newtown, CT, USA) at 40% amplitude, 3 cycles of 2 min each, with 1 min intervals on ice to prevent heating to achieve nanoscale dispersion.

The resulting RES-PNPs were characterized for their critical quality attributes (CQAs), including particle size, polydispersity index (PDI), zeta potential (expected to be negative or near-neutral due to the neutral/anionic nature of phosphatidylcholine and cholesterol in common buffer systems), and encapsulation efficiency (EE%), as these parameters critically determine formulation stability, efficiency, and reproducibility. Transmission electron microscopy (TEM; JEOL JEM-2100, Tokyo, Japan) operated at 200 kV was used to evaluate particle morphology and internal structure. For TEM particle size analysis, at least 100 individual nanoparticles were measured from multiple micrographs using Digital Micrograph (Gatan Microscopy Suite, Version 2.11.1404.0, Gatan Inc., Pleasanton, CA, USA) and SoftImagingSystem software (Version 5.2, Olympus-SIS, Münster, Germany)., and the average size ± standard deviation (SD) was calculated to ensure statistical reliability. Micrographs were digitally processed using Digital Micrograph and SoftImagingSystem software. Dynamic light scattering (DLS) analysis was performed with a Zetasizer Nano ZS (Malvern Instruments, Malvern, UK) to measure hydrodynamic size, PDI, and zeta potential at 25 °C, following appropriate dilution with deionized water to minimize multiple-scattering effects. A magnitude exceeding ±25 mV is indicative of colloidal stability, although the sign of the charge reflects the inherent nature of the formulation components rather than the presence of cationic additives.

Encapsulation efficiency (EE%) was determined by ultracentrifugation at 20,000× *g* for 30 min at 4 °C using a fixed-angle rotor (JA-25.50, Beckman Coulter, Brea, CA, USA) with an 8.5 cm radius; RCF (×*g*) is reported instead of RPM to ensure reproducibility. The free RES content in the supernatant was quantified spectrophotometrically at 257 nm using a validated UV/Vis method. The method was validated by constructing a calibration curve with standard RES solutions (concentration range: 5–50 µg/mL), which showed excellent linearity (R^2^ = 0.998). Precision was confirmed by performing intra-day and inter-day measurements on triplicate samples, yielding relative standard deviations (RSDs) below 2%. EE% was then calculated using the equation: “EE% = [(Total RT − Free RT)/Total RT] × 100” [23].

The optimized RES-PNP formulation was selected based on minimal particle size (<200 nm), PDI < 0.3, high EE (>80%), and a zeta potential magnitude exceeding ±25 mV, collectively indicative of a stable, homogenous, and robust nano-system suitable for biological evaluation.

Fourier-transform infrared (FTIR) spectroscopy was performed to elucidate potential interactions between resveratrol (RES) and lipid components in the phytosomal system. For FTIR sample preparation, the RES-PNPs were first freeze-dried to remove all water, then finely ground and mixed with dry potassium bromide (KBr) at a 1:100 (*w*/*w*) ratio. The mixture was compressed into transparent pellets at room temperature using a hydraulic press, and the pellets were immediately analyzed to prevent moisture absorption. FTIR spectra for pure RES, phosphatidylcholine, their physical mixture, and the optimized RES-PNPs were acquired using a Shimadzu IRTracer-100 spectrometer (Shimadzu Corporation, Kyoto, Japan) in the range of 4000–400 cm^−1^ with a resolution of 4 cm^−1^.

The physical stability of the RES-PNP formulation was examined by storing samples at 4 °C and 25 °C for 30 days. At predetermined intervals (0, 15, and 30 days), particle size, zeta potential, and encapsulation efficiency were measured to assess any changes in formulation attributes. Each measurement was conducted in triplicate, and results were expressed as mean ± standard deviation (SD). Statistical comparisons were performed using one-way ANOVA followed by Tukey’s post hoc test, with *p* < 0.05 considered significant.

### 2.3. Preparation and Viability Determination of EAC Cells

The Ehrlich Ascites Carcinoma cell line was obtained from the “Tumor Biology Department at the National Cancer Institute, Cairo University”. This cell line originated from a spontaneous murine mammary adenocarcinoma that was subsequently adapted into its transplantable ascitic form. The EAC model is widely used owing to its rapid proliferation rate, reproducibility, and ability to generate ascitic tumors upon intraperitoneal inoculation in mice [7]. To maintain tumor drive and ensure consistent biological characteristics, the EAC cell line was serially propagated in vivo via intraperitoneal (i.p.) passage in male Swiss albino mice at 7- to 10-day intervals. All procedures were performed under aseptic conditions. Ascitic fluid was aseptically aspirated from donor mice at peak tumor growth, diluted with sterile normal saline, and used for subsequent inoculations.

For experimental inoculation, EAC cells were collected by gentle aspiration from the peritoneal cavity of tumor-bearing mice. The cell suspension was diluted in sterile normal saline and filtered through a fine-mesh sieve to eliminate tissue debris. Viability and purity of the tumor cell suspension were determined using the Trypan Blue exclusion test, in which nonviable cells absorb the dye while viable cells exclude it. Cell counting was performed using a “Neubauer hemocytometer” under a light microscope.

The viable cell concentration was adjusted to 2.5 × 10^6^ cells per 0.1 mL saline, which was used for intraperitoneal inoculation to induce ascitic tumors in recipient mice. Only cell suspensions with >95% viability were used for experiments to ensure consistent tumor induction and reproducibility.

### 2.4. Animal Care and Experimental Design

Ninety healthy adult male Swiss albino mice (20–25 g each) were obtained from the Animal House, Faculty of Medicine, Mansoura University, Egypt. Animals were housed in sterilized polypropylene cages with stainless-steel mesh lids under standard laboratory conditions (temperature: 24 ± 2 °C; relative humidity: 55%; photoperiod: 12 h light/dark cycle). Standard pellet diet and tap water were provided ad libitum. A one-week acclimatization period preceded the start of experiments to ensure adaptation to the environment.

All experimental procedures were performed in accordance with the Guide for the Care and Use of Laboratory Animals (8th edition, NRC, 2011) and the International Guiding Principles for Biomedical Research Involving Animals (1985). Ethical approval was obtained from the Institutional Ethical Committee (approval number: RC:17.10.2025).

Following acclimatization, the mice were randomly assigned to six groups (n = 15 per group):

• Group 1 (Control): Received only vehicle (distilled water).
• Group 2 (RES): Administered resveratrol (10 mg/kg body weight/day, orally) for 20 days.
• Group 3 (RES-PNPs): Received resveratrol-loaded phytosome nanoparticles (10 mg/kg/day, orally) for 20 days.
• Group 4 (EAC): Inoculated intraperitoneally on day 1 with 0.2 mL of EAC cell suspension containing 2.5 × 106 cells and left untreated for 20 days.
• Group 5 (EAC + RES): EAC-bearing mice treated with RES (10 mg/kg/day, orally) for 20 days post-inoculation.
• Group 6 (EAC + RES-PNPs): EAC-bearing mice treated with RES-PNPs (10 mg/kg/day, orally) for 20 days post-inoculation.

The chosen resveratrol dosage was based on prior pharmacological evidence of efficacy and safety in murine models [24]. The sample size for each experimental group was determined in accordance with contemporary recommendations for preclinical animal research and ARRIVE 2.0 guidelines. A reference to supportive literature and standard power analysis indicates that enrolling 8–12 rodents per group provides 80% statistical power (α = 0.05) to detect meaningful biological differences in key biochemical and histopathological parameters [25]. This group size ensures both statistical robustness and ethical responsibility in accordance with the 3Rs principle (Replacement, Reduction, Refinement) [26]. Selection of the group sizes also reflected a balance between achieving adequate power to discern treatment-related effects across six study groups and minimizing excess animal use. All animals enrolled in the protocol completed it; no subjects or data points were excluded or lost to attrition, thereby supporting the validity of the comparative analyses. All outcome measurements and data analyses in this study were performed in a blinded manner, except for group allocation and animal follow-up, which were necessarily conducted unblinded.

### 2.5. Survival Monitoring and Analysis

Animals were observed daily for survival and clinical signs of distress throughout the study. The mean survival time (MST) was calculated according to the following equation: MST = (first death + last death)/2. The percentage increase in life span (ILS%) for each treatment group was determined using: ILS% = [(NS − C)/C] × 100, where NS denotes the average number of survival days in the treated group, and C represents that of the EAC control group. Prolongation of lifespan is a reliable criterion for assessing anti-tumor efficacy in EAC-bearing mice.

### 2.6. Evaluation of Tumor Growth Parameters

Tumor progression was evaluated based on changes in body weight, ascitic fluid volume, abdominal circumference, and viable tumor cell number. Body weight and abdominal circumference were measured on days 1 and 20 to monitor tumor-associated ascitic development. At the study endpoint, ascitic fluid was collected by gentle aspiration using a 5 mL sterile syringe and its volume quantified in a graduated centrifuge tube as previously described [27].

For viable cell determination, a 9:1 mixture of ascitic fluid and 1% Trypan Blue solution was incubated for 10 min at 37 °C. Live (unstained) and dead (stained) cells were counted within 5 min. The number of viable tumor cells was calculated according to the formula: “Viable cell count = (Number of cells × Dilution factor)/(Area × Thickness of liquid film)” [28]. These quantitative indices provided comparative measures of tumor response to treatment in all experimental groups.

### 2.7. VEGF Analysis in Ascitic Fluid

VEGF levels in ascitic fluid were quantified using a Mouse VEGF Quantikine^®^ ELISA kit (R&D Systems Europe, Ltd., London, UK) according to the manufacturer’s protocol. Ascitic samples were diluted with Calibrator Diluent RD5T, and 50 μL each of diluted sample and Assay Diluent RD1N were added to a pre-coated 96-well plate. Plates were incubated for 2 h at room temperature to permit antigen–antibody binding. After washing, 100 μL of peroxidase-conjugated anti-VEGF antibody was added and incubated for another 2 h. Color development was initiated with the substrate reagent for 30 min and stopped according to the kit guidelines. Absorbance was read spectrophotometrically, and VEGF concentrations were calculated from a standard curve. The ELISA principle follows a sandwich binding configuration, ensuring linear measurement within the pg/mL range.

### 2.8. RNA Extraction and Quantitative Real-Time PCR (qRT-PCR)

Total RNA was extracted from EAC cells harvested from ascitic fluid using the Thermo Scientific GeneJET™ RNA Purification Kit (Thermo Scientific, Waltham, MA, USA) in accordance with the supplied instructions. Five micrograms of purified RNA were reverse-transcribed to cDNA using the RevertAid First Strand cDNA Synthesis Kit (Thermo Scientific, Waltham, MA, USA). Gene-specific primers for *caspase-3*, *Bax*, *Bcl-2*, and *GAPDH* were designed through NCBI Primer3-BLAST and synthesized by Invitrogen (Carlsbad, CA, USA) (Table 1). *GAPDH* served as the reference housekeeping gene. Amplification was performed with a Rotor-Gene Q real-time PCR system (Qiagen, Hilden, Germany), and relative gene expression levels were calculated using the 2^−ΔΔCt^ method [29].

### 2.9. Sample Collection

At the end of the experiment, mice were fasted overnight and anesthetized with isoflurane. Body weights were recorded before sacrifice. Blood samples were collected from the retro-orbital plexus without an anticoagulant, allowed to clot at room temperature, and then centrifuged at 3000× *g* for 15 min to isolate serum for hormonal assays. Following euthanasia, both testes were excised, washed with chilled saline, and processed as follows: one testis was stored at −80 °C for biochemical and molecular analyses, and the counterpart was fixed in 10% neutral-buffered formalin for histopathological and ultrastructural evaluations.

### 2.10. Hormonal Analysis

Hormonal profiling included measurements of luteinizing hormone (LH), follicle-stimulating hormone (FSH), and testosterone. Serum LH levels were determined using an ELISA kit (Biodiagnostic, Dokki, Giza,, Egypt) as described by [30]. Serum FSH concentrations were measured via a double-antibody radioimmunoassay (Diagnostic Products Corporation, Los Angeles, CA, USA) following [31]. At the same time, serum testosterone levels were quantified using a standard diagnostic immunoassay (Diagnostic Products Corporation, Los Angeles, CA, USA) [32].

### 2.11. Antioxidant and Inflammatory Status

Testes were homogenized in ice-cold phosphate buffer (pH 7.4) and centrifuged at 4000 rpm for 15 min (4 °C). The supernatant was used for determining enzymatic and non-enzymatic antioxidant parameters. Colorimetric assays were conducted to estimate the activities of superoxide dismutase (SOD; Cat. No. SD 2521), catalase (CAT; Cat. No. CA 2517), and glutathione peroxidase (GSH-Px; Cat. No. GP 2524), as well as the concentrations of reduced glutathione (GSH; Cat. No. GR 2511). Lipid peroxidation was assessed by quantifying malondialdehyde (MDA; Cat. No. MD 2529) via the TBARS assay, and protein oxidation was quantified by protein carbonyl content (Cat. No. PC 2520) using commercial Biodiagnostic kits. Pro-inflammatory cytokines, including interleukin-1β (IL-1β), tumor necrosis factor-α (TNF-α), and interferon-γ (IFN-γ), were quantified in testicular homogenates using ELISA kits (MyBioSource, San Diego, CA, USA; IL-1β: MBS2881170, TNF-α: MBS3805961, and IFN-γ: MBS9018347), according to the manufacturer-recommended sandwich immunoassay method.

### 2.12. Cyclooxygenase-2 (COX-2) Activity

Testicular COX-2 activity was determined spectrophotometrically. Reaction mixtures (final volume 1 mL) containing purified COX-2 (10%), hematin (60 μM), and EDTA (3 μM) were pre-incubated at 25 °C for 10 min. TMPD (3.3 mM) and arachidonic acid (200 μM) were subsequently added, and the oxidation rate of TMPD was measured at 590 nm. The enzyme activity was calculated based on the extinction coefficient of 0.00826 μM^−1^, where one unit corresponds to oxidation of 1 nmol TMPD/min at 25 °C.

### 2.13. Epididymal Sperm Analysis

The cauda epididymis was excised and minced in 1 mL phosphate-buffered saline (pH 7.2) to obtain a sperm suspension, which was filtered to remove tissue fragments. Sperm counts were performed using a Neubauer hemocytometer, while motility and morphological abnormalities were examined microscopically at 100× magnification under a heated stage [33].

### 2.14. Histopathological and Ultrastructural Examination

For histological evaluation, fixed testes were embedded in paraffin, sectioned at 5 µm thickness, and stained with hematoxylin and eosin. Sections were observed using a light microscope (Olympus CX23, Olympus Corporation, Tokyo, Japan) at 400× magnification. For transmission electron microscopy (TEM), small tissue fragments were fixed in 2.5% glutaraldehyde (24 h, 4 °C), post-fixed with 1% osmium tetroxide (2 h), dehydrated in graded ethanol, cleared in acetone, and embedded in epoxy resin. Ultrathin sections (60–70 nm) were stained with uranyl acetate and lead citrate, and examined under a JEOL JEM-2100 TEM (JEOL Ltd., Tokyo, Japan) operating at 160 kV.

### 2.15. Statistical Analysis

Data normality and homogeneity were evaluated using the Shapiro–Wilk and Levene’s tests, respectively. One-way ANOVA was employed for group comparisons using SAS Proc ANOVA (version 9.3, SAS Institute Inc., Cary, NC, USA), followed by Tukey’s post hoc test. All results were expressed as mean ± SEM, and differences were considered statistically significant at *p* < 0.05. Visual data representations, including bar charts and scatterplots, were generated using GraphPad Prism 9.0.

## 3. Results

### 3.1. Molecular Docking Results

**Figure 1:**
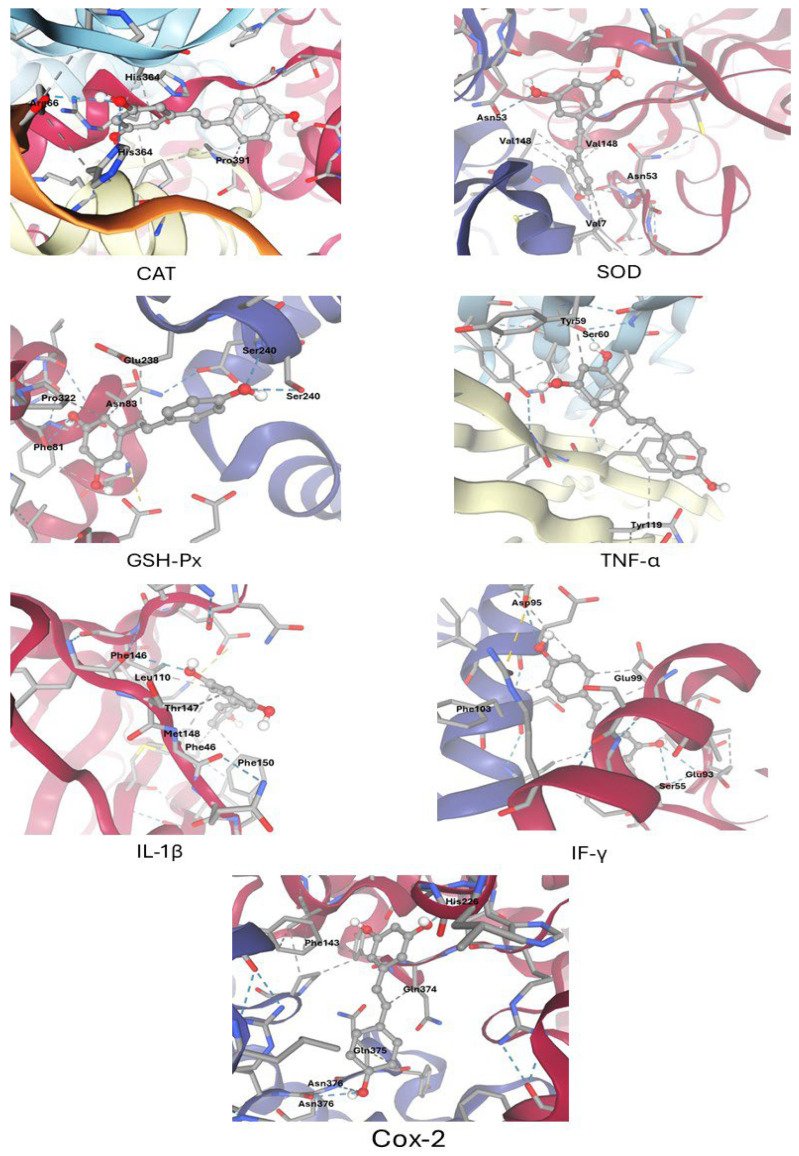
Docking of resveratrol with the key protein involved in oxidative stress and inflammation Pathways [catalase (CAT), superoxide dismutase (SOD), glutathione peroxidase (GSH-Px), tumor necrosis factor-alpha (TNF-α), interleukin-1β (IL-1β), interferon-gamma (IFN-γ), and cyclooxygenase-2 (COX-2)]. Hydrogen bonds and hydrophobic contacts are represented by dotted blue and gray lines, respectively. Key interacting residues are shown in the figure.

Molecular docking simulations revealed that resveratrol exhibits a spectrum of binding affinities toward key antioxidant and pro-inflammatory protein targets, with predicted binding energies ranging from −5.4 to −7.6 kcal/mol (Table 2, Figure 1). Among all targets examined, cyclooxygenase-2 (COX-2) demonstrated the strongest interaction with resveratrol (−7.6 kcal/mol), characterized by the formation of three hydrogen bonds and three hydrophobic contacts involving key residues Asn376, Gln374, Gln375, Phe143, and His228.

Moderately strong binding affinities were observed for catalase (CAT; −6.9 kcal/mol) and tumor necrosis factor-alpha (TNF-α; −6.6 kcal/mol), with specific hydrogen bonds documented at His364 (CAT) and Ser60 (TNF-α), respectively. Superoxide dismutase (SOD; −6.2 kcal/mol) and glutathione peroxidase (GSH-Px; −6.4 kcal/mol) showed several hydrophobic and hydrogen bond interactions with catalytically relevant residues, including Asn53, Pro322, and Ser240. The binding affinities for interferon-gamma (IFN-γ; −6.4 kcal/mol) and interleukin-1β (IL-1β; −5.4 kcal/mol) were lower, with interaction profiles dominated by multiple polar and hydrophobic contacts.

These findings collectively suggest that resveratrol, through favorable binding energies and specific interactions with catalytic and regulatory residues of antioxidant and inflammatory targets, may mechanistically confer both antioxidant and anti-inflammatory activities. Representative docking scores and key interacting residues are summarized in Table 2.

### 3.2. Characterization of Resveratrol-Loaded Phytosome Nanoparticles (RES-PNPs)

**Figure 2:**
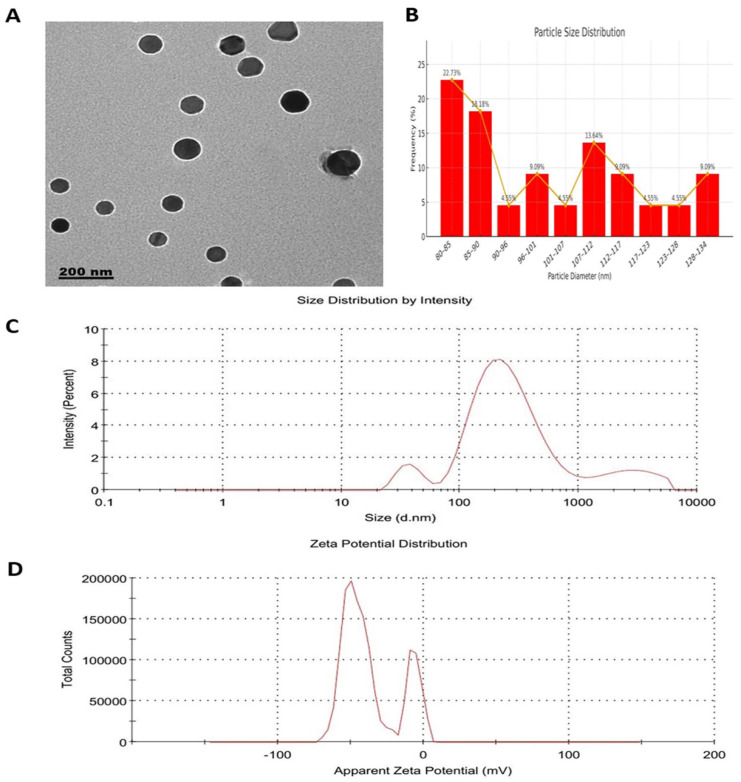
Morphological and physicochemical characterization of resveratrol-loaded phytosome nanoparticles (RES-PNPs). (**A**) Transmission electron microscopy (TEM) micrograph illustrating RES-PNPs with nearly spherical, smooth, and well-defined morphology, confirming uniform vesicle formation and successful encapsulation of resveratrol within the phospholipid matrix. (**B**) Particle size distribution histogram showing nanoparticle diameters predominantly in the 80–135 nm range, indicating a narrow size distribution. (**C**) Dynamic light scattering (DLS) z-average size distribution by volume. (**D**) Zeta potential distribution curve demonstrating high colloidal stability. The average hydrodynamic diameter, polydispersity index (PDI), and zeta potential (ZP) values of the optimized RES-PNPs were 190 nm, 0.486, and −36 mV, respectively, confirming good dispersity and electrostatic stability.

Transmission electron microscopy (TEM) images confirmed that RES-PNPs were spherical, evenly dispersed, and morphologically uniform (Figure 2A). Particle diameters ranged between 80 and 135 nm (Figure 2B), consistent with typical phytosomal nanocarrier dimensions reported previously. Dynamic light scattering (DLS) analysis revealed a mean hydrodynamic diameter of 190 nm and a polydispersity index (PDI) of 0.486, indicating moderate dispersion uniformity (Figure 2C). The zeta potential of −36 mV (Figure 2D) reflected a highly stable nano-system due to sufficient electrostatic repulsion that prevents nanoparticle aggregation. The encapsulation efficiency (EE = 84.03%) confirmed effective entrapment of resveratrol within the phospholipid matrix, thereby improving stability and bioavailability compared with free resveratrol formulations.

**Figure 3:**
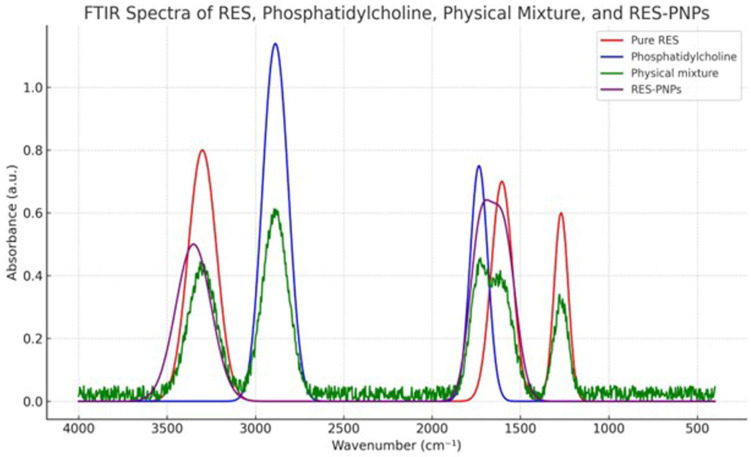
FTIR spectra of pure RES, Phosphatidylcholine, their physical mixture, and the formulated RES-CNPs.

The FTIR spectrum of pure RES displayed characteristic absorption bands at ~3200–3400 cm^−1^ (hydroxyl stretching), 1605 cm^−1^ (aromatic C=C stretching), and 1270 cm^−1^ (C–O stretching in the phenolic structure). Phosphatidylcholine exhibited major bands at 2920 and 2850 cm^−1^ (aliphatic C–H stretching) and 1735 cm^−1^ (ester C=O stretching). In the RES-PNP spectrum, these peaks showed altered intensities and slight shifts, most notably in the –OH and C=O regions, reflecting strong hydrogen bonding and complexation between RES and the lipid matrix. The attenuation and broadening or disappearance of distinctive RES bands further supported effective entrapment of RES within the phytosomal structure, rather than mere physical mixing. Figure 3 depicts the FTIR spectra of each component, their physical mixture, and the formulated RES-PNPs, highlighting the observed shifts and band broadening indicative of nanoparticle formation and interaction. Regarding stability, no significant changes in particle size, zeta potential, or encapsulation efficiency were observed for RES-PNPs stored at either temperature over the 30-day observation period, confirming the physical stability of the optimized formulation.

Long-term stability analysis revealed distinct differences in the physical characteristics of RES-PNPs under various storage conditions (Table 3). Samples stored at 4 °C exhibited no statistically significant changes in particle size, polydispersity index (PDI), zeta potential, or encapsulation efficiency (EE%) over the 30-day storage period. This finding indicates excellent structural stability and sustained drug encapsulation when refrigerated.

In contrast, samples maintained at 25 °C demonstrated significant alterations in key formulation parameters. Specifically, particle size and PDI increased markedly, accompanied by a reduction in both the absolute value of zeta potential and EE%, consistent with particle aggregation and partial leakage of encapsulated resveratrol under ambient conditions. These results suggest that room temperature storage compromises the formulation’s integrity, potentially diminishing its pharmacological effectiveness.

### 3.3. Changes in Tumor Growth and Survival Parameters

As summarized in Table 4, untreated Ehrlich Ascites Carcinoma (EAC)-bearing mice exhibited an MST of 7.5 days. Oral administration of free RES prolonged survival to 11 days, while RES-PNP treatment extended it further to 12 days, representing 46.67% and 60.0% increases in lifespan, respectively. EAC inoculation caused significant ascitic fluid accumulation and elevated body weight and abdominal circumference. Both RES and RES-PNPs markedly reduced ascites volume, with a more pronounced reduction in the RES-PNPs group. Similarly, increases in body weight and abdominal circumference in the EAC group were significantly mitigated by both treatments, and the RES-PNPs formulation achieved superior suppression. Viable tumor cell counts declined significantly in treated groups; notably, RES-PNPs produced a stronger cytotoxic response than free RES. These modifications collectively highlight the enhanced anti-tumor efficacy of nanoparticle-encapsulated RES compared with its non-encapsulated form.

### 3.4. Expression Profiles of Apoptotic-Regulatory Genes

**Figure 4:**
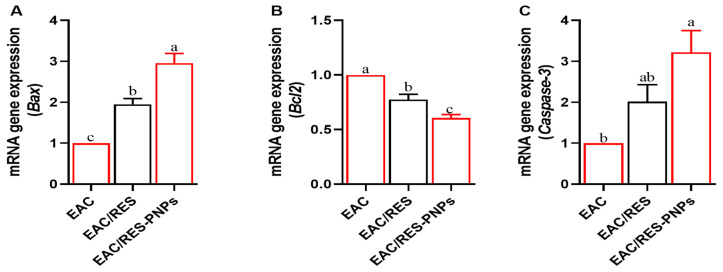
Changes in Expression profiles of (**A**) *Bax*, (**B**) *Bcl-2*, and (**C**) *Caspase-3* genes in Ehrlich Ascites Carcinoma (EAC)-bearing mice treated with resveratrol-loaded phytosome nanoparticles and free resveratrol. Bax: Bcl-2-associated X protein; Bcl-2: B-cell lymphoma-2; Caspase-3: Cysteine-dependent Aspartate-directed Protease-3. EAC: Inoculated with Ehrlich Ascites Carcinoma (EAC) cells (0.2 mL); EAC/RES: resveratrol (10 mg/kg body weight) + EAC cells (0.2 mL); EAC/RES-PNPs: resveratrol-loaded phytosome nanoparticles (10 mg/kg body weight) + EAC cells (0.2 mL) (each group n = 10). Data are expressed as mean ± SE. Values bearing different superscript letters (a, b, c) differ significantly at *p* < 0.05.

Quantitative PCR analyses (Figure 4A–C) demonstrated significant upregulation of the pro-apoptotic genes *Bax* and *Caspase-3* in both RES and RES-PNP groups compared with the EAC control. The RES-PNPs treatment produced higher expression levels of these apoptosis-related markers than free RES. Conversely, Bcl-2 mRNA, the anti-apoptotic gene, was markedly elevated in the untreated EAC group, but significantly suppressed following both treatments, with the RES-PNPs formulation achieving greater downregulation. This reciprocal modulation of *Bax/Caspase-3* activation and *Bcl-2* suppression confirms that RES-PNPs potentiate apoptotic signaling within tumor cells more effectively than free RES.

### 3.5. VEGF Levels in Ascitic Fluid

**Figure 5:**
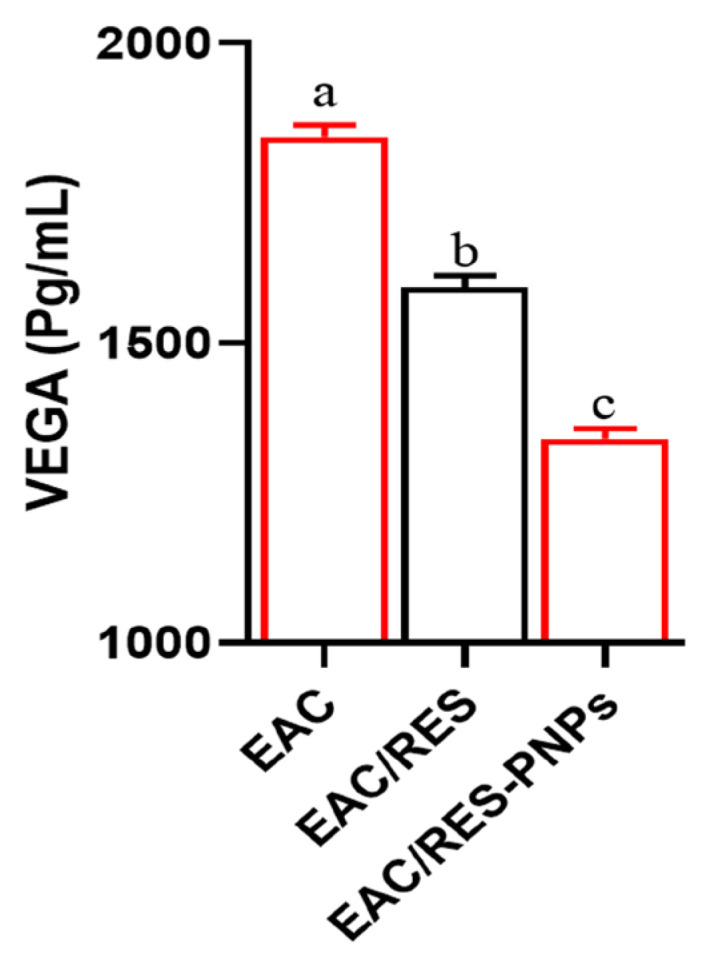
Modulation of Vascular Endothelial Growth Factor in Ascitic Fluid of Ehrlich Ascites Carcinoma-Bearing Mice Treated with Resveratrol-Loaded Phytosome Nanoparticles and Free Resveratrol. RES: resveratrol (10 mg/kg body weight); RES-PNPs: resveratrol-loaded phytosome nanoparticles (10 mg/kg body weight); EAC: Inoculated with Ehrlich Ascites Carcinoma (EAC) cells (0.2 mL); EAC/RES: resveratrol (10 mg/kg body weight) + EAC cells (0.2 mL); EAC/RES-PNPs: resveratrol-loaded phytosome nanoparticles (10 mg/kg body weight) + EAC cells (0.2 mL) (each group n = 10). Data are expressed as mean ± SE. Values bearing different superscript letters (a, b, c) differ significantly at *p* < 0.05.

EAC induction led to significantly elevated VEGF levels in ascitic fluid, reflecting pronounced angiogenic stimulation (Figure 5). Treatment with free RES significantly reduced VEGF concentrations; however, RES-PNPs achieved a more profound suppression, demonstrating the lowest VEGF values among all groups.

### 3.6. Semen Quality

As shown in Table 5, sperm count and motility were significantly decreased in EAC-bearing mice compared with control groups. Both RES and RES-PNPs treatments improved these parameters, restoring sperm quality substantially. The RES-PNPs group exhibited the highest count and motility, approaching those of non-tumor controls. Meanwhile, abnormal sperm morphology increased significantly in untreated EAC animals but declined markedly after treatment, with RES-PNPs producing the lowest abnormality rate.

### 3.7. Hormonal Profile

EAC inoculation resulted in marked suppression of serum testosterone, LH, and FSH concentrations compared with all control and treatment groups (Table 6). Treatment with either free RES or RES-PNPs significantly restored these hormone levels. The elevation was most pronounced in the RES-PNPs group, which achieved testosterone and LH levels comparable to those in the normal control group. In contrast, free RES partially restored hormone levels but remained significantly lower than those of the RES-PNPs group.

### 3.8. Redox Status in Testicular Tissue

Table 7 presents the oxidative and antioxidant indices of testicular tissue. The EAC group demonstrated substantial decreases in enzymatic antioxidants (SOD, CAT, GSH-Px) and GSH levels, together with significant elevations in oxidative damage markers (MDA and protein carbonyl). Administration of both RES and RES-PNPs restored antioxidant enzyme activities and reduced oxidative stress biomarkers, with RES-PNPs exerting a stronger protective effect. Antioxidant values in the RES-PNPs group were statistically comparable to control values. Similarly, MDA levels in the RES-PNPs group were indistinguishable from those of normal controls, confirming the remarkable antioxidant potency of RES-PNPs.

### 3.9. Testicular Inflammation and COX-2 Activity

**Figure 6:**
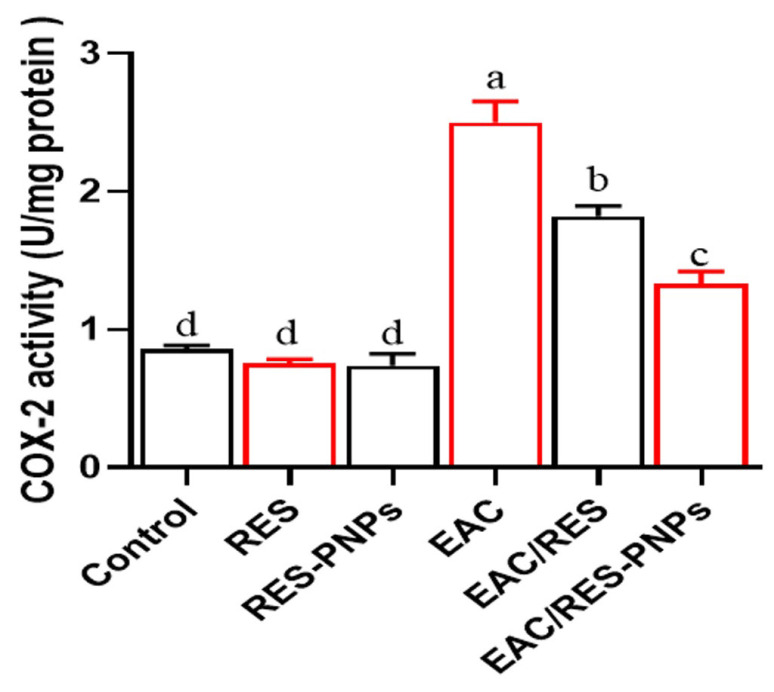
Cyclooxygenase-2 (COX-2) activity in testicular tissue of Ehrlich ascites carcinoma-bearing mice treated with resveratrol-loaded phytosome nanoparticles and free resveratrol. RES: resveratrol (10 mg/kg body weight); RES-PNPs: resveratrol-loaded phytosome nanoparticles (10 mg/kg body weight); EAC: Inoculated with Ehrlich Ascites Carcinoma (EAC) cells (0.2 mL); EAC/RES: resveratrol (10 mg/kg body weight) + EAC cells (0.2 mL); EAC/RES-PNPs: resveratrol-loaded phytosome nanoparticles (10 mg/kg body weight) + EAC cells (0.2 mL) (each group n = 10). Data are expressed as mean ± SE. Values bearing different superscript letters (a, b, c, d) differ significantly at *p*< 0.05.

**Figure 7:**
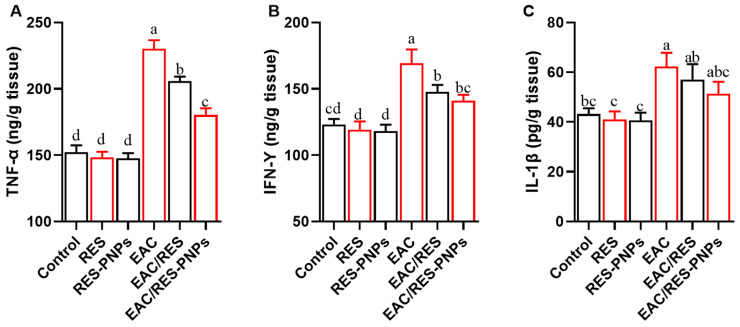
Changes in Inflammatory Cytokines in Testicular Tissue of Ehrlich Ascites Carcinoma-Bearing Mice Treated with Resveratrol-Loaded Phytosome Nanoparticles and Free Resveratrol. TNF-α: Tumor Necrosis Factor-alpha (**A**); INF-γ: Interferon-gamma (**B**); IL-1β: Interleukin-1 beta (**C**). RES: resveratrol (10 mg/kg body weight); RES-PNPs: resveratrol-loaded phytosome nanoparticles (10 mg/kg body weight); EAC: Inoculated with Ehrlich Ascites Carcinoma (EAC) cells (0.2 mL); EAC/RES: resveratrol (10 mg/kg body weight) + EAC cells (0.2 mL); EAC/RES-PNPs: resveratrol-loaded phytosome nanoparticles (10 mg/kg body weight) + EAC cells (0.2 mL) (each group n = 10). Data are expressed as mean ± SE. Values bearing different superscript letters (a, b, c, d) differ significantly at *p* < 0.05.

COX-2 activity was markedly increased in EAC-bearing mice (Figure 6), confirming an upregulated inflammatory milieu. Both RES and RES-PNP treatments significantly reduced COX-2 enzymatic activity, with the lowest values observed in the RES-PNPs group. The pro-inflammatory cytokines TNF-α, IFN-γ, and IL-1β were significantly elevated following tumor induction (Figure 7A–C). Both treatments ameliorated this response, yet cytokine levels in the RES-PNPs-treated group returned to near baseline levels, with no significant difference from the control group.

### 3.10. Histopathological Observations: Ultrastructural Alterations

**Figure 8:**
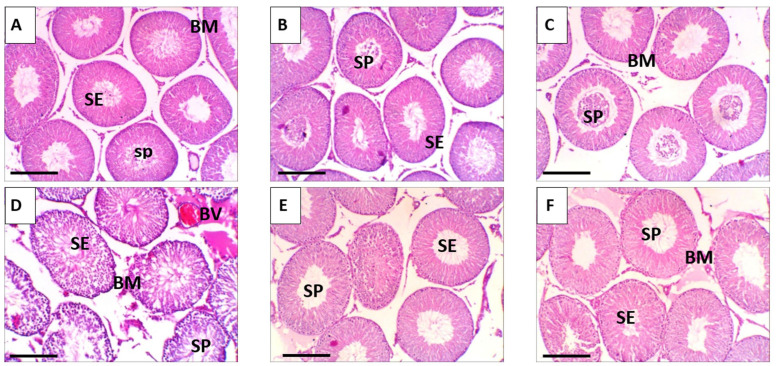
Representative photomicrographs of testicular sections from the control and experimental groups. (**A**) Control group; (**B**) RES: resveratrol (10 mg/kg body weight); (**C**) RES-PNPs: resveratrol-loaded phytosome nanoparticles (10 mg/kg body weight); (**D**) EAC: mice inoculated with Ehrlich Ascites Carcinoma (EAC) cells (0.2 mL); (**E**) EAC/RES: resveratrol (10 mg/kg body weight) + EAC cells (0.2 mL); (**F**) EAC/RES-PNPs: resveratrol-loaded phytosome nanoparticles (10 mg/kg body weight) + EAC cells (0.2 mL). Abbreviations: BM, basement membrane; SP, spermatozoa; SE, seminiferous epithelium. All images were captured at 400× magnification; scale bar = 50 µm.

Histological evaluation of testicular tissues in the various experimental groups revealed pronounced differences in morphological integrity. In the control group, as well as in mice treated with free RES or RES-PNPs, the testes exhibited normal histoarchitecture, with well-organized seminiferous tubules, intact germinal epithelium, and preserved spermatogenic series (Figure 8A–C). Spermatocytes and supporting Sertoli cells maintained normal spatial relationships, and germ cell maturation was evident throughout the seminiferous layers. In contrast, the EAC-bearing group displayed extensive architectural disruption characterized by widespread detachment of germinal epithelial cells, fragmentation of the seminiferous epithelium, and loss of orderly spermatogenic stratification (Figure 8D). Notable nuclear alterations, including karyorrhexis and pyknosis, were frequently observed, indicative of ongoing apoptotic processes and EAC-induced cytotoxic injury. Treatment of EAC-bearing mice with either free RES or RES-PNPs substantially ameliorated these histopathological changes. In both groups, the overall testicular structure remained largely intact, with restoration of seminiferous tubule organization and germ cell alignment. Occasional vacuolation within spermatogenic cell cytoplasm and mild thinning of the spermatogenic layer were observed, yet germinal epithelium integrity and germ cell maturation capacity were preserved to a significant extent (Figure 8E,F).

### 3.11. Ultrastructural Alterations

**Figure 9:**
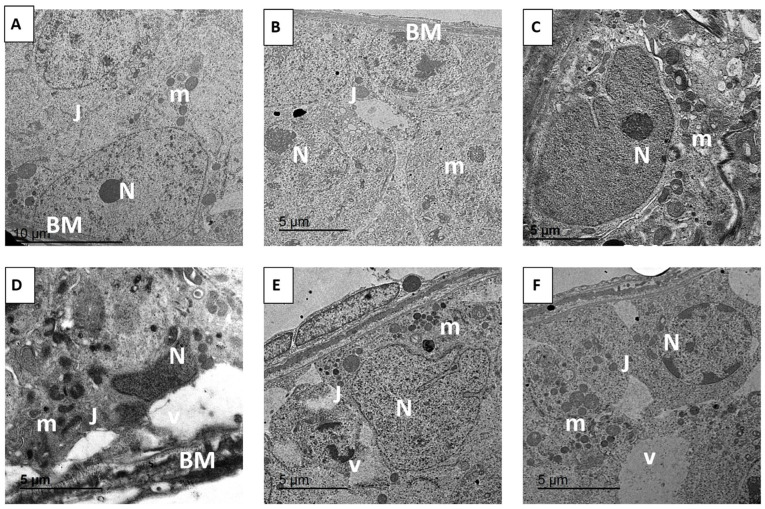
Representative ultrastructural photomicrographs of testicular tissue from the control and experimental groups. (**A**) Control group; (**B**) RES: resveratrol (10 mg/kg body weight); (**C**) RES-PNPs: resveratrol-loaded phytosome nanoparticles (10 mg/kg body weight); (**D**) EAC: mice inoculated with Ehrlich Ascites Carcinoma (EAC) cells (0.2 mL); (**E**) EAC/RES: resveratrol (10 mg/kg body weight) + EAC cells (0.2 mL); (**F**) EAC/RES-PNPs: resveratrol-loaded phytosome nanoparticles (10 mg/kg body weight) + EAC cells (0.2 mL). Abbreviations: N, nucleus; m, mitochondria; S, Sertoli cells; V, cytoplasmic vacuolation; J, junctions of the blood–testis barrier; BM, basement membrane.

Transmission electron microscopy provided detailed insights into the subcellular changes in testicular tissue following EAC induction and subsequent treatment with free RES or RES-PNPs (Figure 9). In the control, RES-treated, and RES-PNPs-treated groups (Figure 9A–C), seminiferous tubules exhibited normal ultrastructural organization with an intact basement membrane and well-preserved germinal epithelium. Spermatogonia displayed large oval nuclei rich in euchromatin, abundant mitochondria with intact cristae, and clearly defined Golgi complexes. Sertoli cells showed regular boundaries with prominent tight junctions, reflecting a stable blood-testis barrier. Leydig cells within the interstitial compartment contained normal smooth endoplasmic reticulum and lipid droplets, consistent with preserved steroidogenic activity. In contrast, testes from EAC-bearing mice (Figure 9D) revealed profound ultrastructural degeneration. The seminiferous epithelium was markedly disorganized, with sloughing of germinal cells into the lumina and widespread cellular fragmentation. Mitochondrial swelling, disrupted membranes, and loss of cristae were evident in spermatogenic and Sertoli cells, indicating severe oxidative and apoptotic damage. Chromatin condensation, nuclear pyknosis, and apoptotic bodies were frequently observed, accompanied by the formation of cytoplasmic vacuoles and breakdown of tight junction integrity, signifying collapse of the blood–testis barrier.

Treatment of EAC-bearing mice with RES or RES-PNPs markedly attenuated these degenerative features (Figure 9E,F). The seminiferous tubules exhibited restored organization, and most germinal cells retained normal nuclear morphology and cytoplasmic organelles. Mitochondria regained their normal lamellar cristae and distribution, and Sertoli–germ cell junctional complexes reappeared, re-establishing the barrier function. Notably, RES-PNP treatment resulted in more extensive recovery, with minimal residual vacuolation and nearly complete structural restoration of the spermatogenic layers.

## 4. Discussion

The development of malignant ascites represents a critical event in advanced cancer progression, characterized by excessive peritoneal fluid accumulation, aggressive angiogenesis, and poor clinical outcomes, with median survival rarely exceeding 20 weeks after diagnosis [5]. The EAC model remains an established and biologically relevant system for mimicking the pathophysiological hallmarks of peritoneal carcinomatosis, including uncontrolled cell proliferation, elevated vascular permeability, and high VEGF [7]. As highlighted by the current study, EAC induced angiogenesis, oxidative imbalance, and the release of inflammatory cytokines, processes that were effectively mitigated by RES-PNP treatment.

Nanotechnology-driven delivery systems have demonstrated considerable promise in overcoming the pharmacokinetic limitations of polyphenols, such as resveratrol, including poor aqueous solubility, low stability, and first-pass metabolism, thereby enhancing absorption, bioavailability, and therapeutic efficacy. Phytosomes, in particular, form stable lipid bilayer complexes that facilitate both lipophilic and hydrophilic transport across biological membranes, improving cellular uptake and sustained circulation profiles [22]. In this study, encapsulation of resveratrol into phytosome nanoparticles not only stabilized the compound but also significantly enhanced its anti-tumor, antioxidant, anti-angiogenic, and anti-inflammatory activities compared to its native form.

VEGF signaling plays an indispensable role in tumor-associated angiogenesis, and its overexpression directly contributes to the formation of malignant ascites via increased vascular permeability and endothelial proliferation [34]. The present findings revealed that RES-PNPs markedly suppressed VEGF production in ascitic fluid, indicating potent inhibition of tumor-driven angiogenesis. This observation aligns with recent reports describing resveratrol-mediated downregulation of VEGF through the inhibition of MAPK phosphorylation and modulation of PI3K/Akt/mTOR cascades [35]. In line with these results, nano-resveratrol systems have been shown to enhance anti-angiogenic potency, as observed in colon and breast cancer models where nanoparticle-encapsulated RES demonstrated reduced tumor neovascularization and improved bioavailability [36,37].

The apoptotic findings of this study further corroborate the mechanistic efficacy of RES-PNPs. Enhanced *Bax* and *Caspase-3* expression coupled with decreased *Bcl-2* mRNA levels in EAC-bearing mice indicate activation of intrinsic apoptotic pathways. These gene expression changes mirror those observed in prior investigations, demonstrating that nanoparticle-encapsulated RES induces mitochondrial-mediated apoptosis through cytochrome c release, caspase activation, and regulation of reactive oxygen species [38,39]. Notably, RES-PNPs showed superior apoptotic efficacy compared with free RES, likely due to improved intracellular delivery and retention, a finding consistent with previous reports showing that resveratrol lipid nanoparticles outperform unencapsulated resveratrol in modulating apoptotic gene expression [40].

Beyond tumor control, the current study demonstrated a pronounced protective effect of RES-PNPs against testicular dysfunction induced by EAC-mediated oxidative stress and inflammation. Malignant diseases are known to disrupt reproductive homeostasis by causing oxidative injury in reproductive tissues [8,9]. In the current experiment, EAC-bearing mice exhibited diminished sperm motility, elevated abnormal sperm morphology, and decreased testosterone, LH, and FSH levels, findings consistent with systemic oxidative stress and impaired inflammatory signaling, both of which are known to impair spermatogenesis [41]. Treatment with RES-PNPs significantly reversed these alterations, restoring both hormone concentrations and sperm quality to near-normal levels. These outcomes reiterate resveratrol’s capacity to modulate androgenic signaling, stimulate spermatogenic cell differentiation, and maintain mitochondrial function through SIRT-1 and AMPK activation [42,43].

Mechanistically, EAC induction enhanced lipid peroxidation products (MDA) and protein carbonyl content, along with depleting endogenous antioxidants (SOD, CAT, GSH-Px, and GSH). RES-PNPs treatment counterbalanced these effects, significantly restoring enzymatic activity and antioxidant reserves more effectively than free RES. The suppression of oxidative damage and concomitant enhancement of antioxidative defenses by RES-PNPs may be attributed to the tri-hydroxy stilbene structure of resveratrol, whose reactive hydroxyl moieties efficiently scavenge radicals and stabilize membrane lipids [44,45]. The ability of RES-PNPs to inhibit pro-inflammatory cytokines (TNF-α, IL-1β, and IFN-γ) further highlights the interplay between their antioxidant and anti-inflammatory actions. Moreover, the significant downregulation of COX-2 activity observed in the RES-PNPs group suggests direct inhibition of prostaglandin synthesis and attenuation of NF-κB signaling pathways, in accordance with established mechanisms of resveratrol in cancer prevention [46,47,48]. Collectively, these findings strengthen the notion that RES-PNPs act through an integrated multi-target mechanism that combines apoptosis promotion, ROS scavenging, angiogenesis inhibition, and inflammation suppression.

Recent studies have highlighted the unique mechanisms through which nanoparticles enhance drug efficacy. Nanocarriers such as RES-PNPs can improve solubility, protect active compounds from rapid metabolism, and facilitate targeted delivery to tissues via enhanced cellular uptake and passive accumulation [49]. These properties likely contribute to the observed synergistic anticancer and testicular-protective effects in the current study by increasing local bioavailability, prolonging drug release, and modulating oxidative and inflammatory pathways. Incorporating such mechanistic insights helps to contextualize the enhanced therapeutic performance of RES-PNPs beyond the intrinsic activity of resveratrol alone.

### Study Limitations and Future Directions

Although this study demonstrates the therapeutic potential of RES-PNPs in suppressing EAC progression and protecting testicular function, several limitations should be noted. Mechanistic insights were inferred from gene expression and biochemical assays, without direct in vivo confirmation of key signaling pathways such as PI3K/Akt or NF-κB. Pharmacokinetic and biodistribution data were not obtained, leaving tissue-specific uptake and bioavailability unverified. Comparisons were limited to free RES, and long-term safety, reproductive recovery, and fertility outcomes were not assessed. Additionally, the study did not include an EAC control group receiving empty phytosomes, which prevents ruling out any potential biological effects of the lipid carrier itself. Furthermore, the EAC model represents an aggressive, artificial tumor system that does not fully replicate human solid tumors, and translating these findings into clinical oncology remains limited by species-specific differences in metabolism and nanoparticle delivery. Future studies should address these gaps, including detailed pharmacokinetic profiling, mechanistic validation via proteomic or Western blot analyses, evaluation of alternative nano-platforms, and assessment of translational potential in combination therapies and humanized tumor models.

## 5. Conclusions

In conclusion, RES-PNPs exerted potent anti-tumor and cytoprotective activities in the EAC model. The formulation significantly curtailed tumor growth, lowered ascitic volume, and enhanced mean survival time via coordinated regulation of apoptotic (Bax, Bcl-2, Caspase-3) and angiogenic (VEGF) pathways. Furthermore, RES-PNPs mitigated oxidative and inflammatory stress, normalized reproductive hormones, and restored sperm function more effectively than free resveratrol. Histopathological and ultrastructural analyses confirmed these findings by revealing preserved seminiferous architecture and cellular integrity.

These comprehensive results affirm that RES-PNPs represent a promising nanoscale therapeutic platform that optimally harnesses resveratrol’s multi-target pharmacology for the management of malignant ascites and protection of male reproductive health. Further translational studies and pharmacodynamic investigations are encouraged to confirm their efficacy and safety across broader cancer and toxicological settings.

## Figures and Tables

**Table 1:** Primers and Their Sequences for qRT-PCR.

Gene	Forward Primer 5′ → 3′	Reverse Primer 5′ → 3′
*caspase-3*	GGGGAGCTTGGAACGCTAAG	GAGTCCACTGACTTGCTCCC
*bax*	CTGGATCCAAGACCAGGGTG	GTGAGGACTCCAGCCACAAA
*bcl-2*	GAACTGGGGGAGGATTGTGG	GCATGCTGGGGCCATATAGT
*GAPDH*	AAATGAGAGAGGCCCAGCTAC	GAGGGCTGCAGTCCGTATTTA

*Caspase-3*: Cysteine-aspartic acid protease-3; *Bax*: Bcl-2-associated X protein; *Bcl-2*: B-cell lymphoma 2; *GAPDH*: Glyceraldehyde-3-phosphate dehydrogenase.

**Table 2:** Docking data of resveratrol with the key protein involved in oxidative stress and inflammation Pathways.

Target Proteins	Binding Affinity (Kcal/M)	Hydrogen Bonds	Hydrophobic Contacts	Key Residues
CAT	−6.9	2	2	Arg66, His364(2), Pro391
SOD	−6.2	1	6	Asn53(2),Val148(3), Val7(2)
GSH-Px	−6.4	3	3	Phe81, Pro322, Asn83, Glu238, Ser240(2)
TNF-α	−6.6	1	2	Tyr59, Ser60, Tyr119
IL-1β	−5.4	1	5	Phe46, Phe146, Phe150, Leu110, Ther147, Met148
IF-γ	−6.4	3	4	Asp95 (2), Glu99(2), Glu93, Ser55, Phe103
Cox-2	−7.6	3	3	Asn376(2), Gln375, Gln374, Phe143, His228

CAT: catalase, SOD: superoxide dismutase, GSH-Px: glutathione peroxidase, TNF-α: tumor necrosis factor-alpha, IL-1β: interleukin-1beta, IFN-γ: interferon-gamma, and COX-2: cyclooxygenase-2.

**Table 3:** Changes in particle size, PDI, zeta potential, and encapsulation efficiency (EE%) of RES-PNPs during storage at different temperatures.

Time (Days)	Particle Size	PDI	Zeta Potential	Encapsulation Efficiency
Storage at 4 °C				
0	190.01 ± 4.15	0.486 ± 0.02	−36.0 ± 1.24	84.0 ± 2.01
15	192.63 ± 5.01	0.493 ± 0.01	−35.4 ± 1.16	83.3 ± 2.14
30	197.42 ± 4.33	0.502 ± 0.03	−35.1 ± 1.09	83.6 ± 1.76
Storage at 25 °C				
0	190.01 ± 4.15	0.486 ± 0.02	−36.0 ± 1.24	84.0 ± 2.01
15	214.26 ± 5.12	0.522 ± 0.01	−31.24 ± 1.36	78.26 ± 2.41
30	246.29 ± 6.01	0.549 ± 0.01	−28.36 ± 1.23	72.21 ± 2.35

**Table 4:** Effects of resveratrol-loaded phytosome nanoparticles and free resveratrol on survival time, and tumor growth response parameters in Ehrlich ascites carcinoma-bearing mice.

Parameters	EAC	EAC/RES	EAC/RES-PNPs
Mean survival time (days)	7.5	11	12
Increased life span percentage (%)	--	46.67	60.00
Change in body weight (%)	42.15 ± 2.14 ^a^	35.52 ± 2.93 ^ab^	28.64 ± 3.29 ^b^
Abdominal circumference (mm)	10.11 ± 0.84 ^a^	8.81 ± 0.72 ^ab^	7.13 ± 0.53 ^b^
Ascitic fluid volume (mL)	8.76 ± 0.37 ^a^	5.19 ± 0.22 ^b^	3.37 ± 0.26 ^c^
Viable EAC cells count (10^6^/mL)	33.27 ± 1.77 ^a^	21.17 ± 1.03 ^b^	12.43 ± 0.84 ^c^

Data are expressed as mean ± SE. RES: resveratrol (10 mg/kg body weight); RES-PNPs: resveratrol-loaded phytosome nanoparticles (10 mg/kg body weight); EAC: Inoculated with Ehrlich Ascites Carcinoma (EAC) cells (0.2 mL); EAC/RES: resveratrol (10 mg/kg body weight) + EAC cells (0.2 mL); EAC/RES-PNPs: resveratrol-loaded phytosome nanoparticles (10 mg/kg body weight) + EAC cells (0.2 mL) (each group n = 15). Values in the same row with different superscript letters (a, b, c) differ significantly at *p* < 0.05.

**Table 5:** Effects of Resveratrol-loaded phytosome nanoparticles and free resveratrol on sperm quality parameters in Ehrlich ascites carcinoma-bearing mice.

TRT	Sperm Count (×10^6^)	Sperm Motility (%)	Sperm Abnormalities (%)
Control	10.12 ± 0.86 ^a^	81.42 ± 2.13 ^ab^	3.57 ± 0.19 ^d^
RES	10.96 ± 0.94 ^a^	83.16 ± 3.11 ^ab^	3.16 ± 0.23 ^d^
RES-PNPs	11.14 ± 1.01 ^a^	84.45 ± 3.86 ^a^	2.88 ± 0.11 ^d^
EAC	6.24 ± 0.43 ^c^	52.32 ± 2.54 ^d^	16.29 ± 1.25 ^a^
EAC/RES	7.15 ± 0.59 ^bc^	63.34 ± 2.17 ^c^	12.37 ± 1.12 ^b^
EAC/RES-PNPs	8.93 ± 0.62 ^ab^	74.12 ± 3.29 ^b^	9.87 ± 0.87 ^c^

Data are expressed as mean ± SE. RES: resveratrol (10 mg/kg body weight); RES-PNPs: resveratrol-loaded phytosome nanoparticles (10 mg/kg body weight); EAC: Inoculated with Ehrlich Ascites Carcinoma (EAC) cells (0.2 mL); EAC/RES: resveratrol (10 mg/kg body weight) + EAC cells (0.2 mL); EAC/RES-PNPs: resveratrol-loaded phytosome nanoparticles (10 mg/kg body weight) + EAC cells (0.2 mL) (each group n = 10). Values in the same column with different superscript letters (a, b, c, d) differ significantly (*p* < 0.05).

**Table 6:** Effects of resveratrol-loaded phytosome nanoparticles and free resveratrol on reproductive hormones in Ehrlich ascites carcinoma-bearing mice.

TRT	Testosterone (ng/mL)	LH (ng/mL)	FSH (ng/mL)
Control	3.22 ± 0.21 ^a^	6.07 ± 0.41 ^a^	5.14 ± 0.33 ^a^
RES	3.41 ± 0.29 ^a^	6.12 ± 0.38 ^a^	5.19 ± 0.35 ^a^
RES-PNPs	3.54 ± 0.18 ^a^	6.33 ± 0.53 ^a^	5.32 ± 0.42 ^a^
EAC	1.12 ± 0.08 ^c^	3.26 ± 0.18 ^b^	1.86 ± 0.05 ^c^
EAC/RES	1.83 ± 0.07 ^b^	4.12 ± 0.22 ^b^	2.17 ± 0.09 ^c^
EAC/RES-PNPs	2.24 ± 0.12 ^b^	5.37 ± 0.26 ^a^	3.11 ± 0.17 ^b^

Data are expressed as mean ± SE. LH: luteinizing hormone; FSH: follicle-stimulating hormone; RES: resveratrol (10 mg/kg body weight); RES-PNPs: resveratrol-loaded phytosome nanoparticles (10 mg/kg body weight); EAC: Inoculated with Ehrlich Ascites Carcinoma (EAC) cells (0.2 mL); EAC/RES: resveratrol (10 mg/kg body weight) + EAC cells (0.2 mL); EAC/RES-PNPs: resveratrol-loaded phytosome nanoparticles (10 mg/kg body weight) + EAC cells (0.2 mL) (each group n = 10). Values in the same column with different superscript letters (a, b, c) differ significantly (*p* < 0.05).

**Table 7:** Effects of Resveratrol-Loaded Phytosome Nanoparticles and Free Resveratrol on Redox Status in Testicular Tissue of Ehrlich Ascites Carcinoma-Bearing Mice.

Parameters	SOD (U/mg protein)	CAT (U/mg protein)	GSH-Px (U/mg protein)	GSH (nmol/g protein)	PC (nmol/g protein)	MDA (nmol/g tissue)
Control	8.41 ± 0.45 ^ab^	19.32 ± 1.12 ^ab^	14.12 ± 0.75 ^bc^	10.12 ± 0.63 ^a^	2.14 ± 0.12 ^d^	21.12 ± 1.33 ^cd^
RES	9.13 ± 0.71 ^a^	20.12 ± 1.39 ^a^	15.32 ± 0.83 ^ab^	10.69 ± 0.54 ^a^	1.95 ± 0.11 ^d^	20.12 ± 1.52 ^d^
RES-PNPs	9.83 ± 0.62 ^a^	21.95 ± 1.62 ^a^	16.47 ± 0.66 ^a^	10.95 ± 0.46 ^a^	1.84 ± 0.29 ^d^	19.41 ± 1.32 ^d^
EAC	3.16 ± 0.13 ^d^	7.16 ± 0.55 ^c^	6.69 ± 0.42 ^d^	4.82 ± 0.28 ^c^	4.85 ± 0.22 ^a^	35.96 ± 2.94 ^a^
EAC/RES	4.91 ± 0.21 ^c^	11.13 ± 0.93 ^c^	8.16 ± 0.51 ^d^	5.11 ± 0.33 ^c^	3.47 ± 0.23 ^b^	31.12 ± 3.11 ^ab^
EAC/RES-PNPs	7.49 ± 0.44 ^b^	15.93 ± 0.99 ^b^	12.74 ± 0.73 ^c^	8.14 ± 0.53 ^b^	2.75 ± 0.12 ^c^	27.93 ± 2.39 ^bc^

Data are expressed as mean ± SE. SOD: superoxide dismutase; CAT: catalase; GSH-Px: glutathione peroxidase; GSH: glutathione; PC: protein carbonyle; MDA: malondialdehyde; RES: resveratrol (10 mg/kg body weight); RES-PNPs: resveratrol-loaded phytosome nanoparticles (10 mg/kg body weight); EAC: Inoculated with Ehrlich Ascites Carcinoma (EAC) cells (0.2 mL); EAC/RES: resveratrol (10 mg/kg body weight) + EAC cells (0.2 mL); EAC/RES-PNPs: resveratrol-loaded phytosome nanoparticles (10 mg/kg body weight) + EAC cells (0.2 mL) (each group n = 10). Values in the same column with different superscript letters (a, b, c, d) differ significantly (*p* < 0.05).

## Data Availability

The original contributions presented in this study are included in the article. Further inquiries can be directed to the corresponding author.

## Abbreviations

The following abbreviations are used in this manuscript:

RES	Resveratrol
RES-PNPs	Resveratrol-loaded phytosome nanoparticles
EAC	Ehrlich Ascites Carcinoma
CAT	Catalase
SOD	Superoxide dismutase
GSH-Px	Glutathione peroxidase
TNF-α	Tumor necrosis factor-alpha
IL-1β	Interleukin-1 beta
IFN-γ	Interferon-gamma
COX-2	Cyclooxygenase-2
TEM	Transmission electron microscopy
DLS	Dynamic light scattering
PDI	Polydispersity index
EE or EE%	Encapsulation efficiency
DMSO	Dimethyl sulfoxide
PBS	Phosphate-buffered saline
MST	Mean survival time
ILS%	Percentage increase in lifespan
VEGF	Vascular endothelial growth factor
qRT-PCR	(Quantitative) real-time polymerase chain reaction
GAPDH	Glyceraldehyde-3-phosphate dehydrogenase
LH	Luteinizing hormone
FSH	Follicle-stimulating hormone
GSH	Glutathione
MDA	Malondialdehyde
PC	Protein carbonyl
FTIR	Fourier-transform infrared spectroscopy
KBr	Potassium bromide
SD	Standard deviation
SE	Standard error
ANOVA	Analysis of variance
H&E	Hematoxylin and eosin (stain)
MAPK	Mitogen-activated protein kinase
PI3K	Phosphoinositide 3-kinase
Akt	Protein kinase B (Akt)
mTOR	Mammalian target of rapamycin
AMPK	AMP-activated protein kinase
SIRT-1	Sirtuin 1
N/A	Not applicable
SEM	Standard error of the mean
HPLC	High-performance liquid chromatography
